# Lineage-specific variations of congruent evolution among DNA sequences from three genomes, and relaxed selective constraints on *rbc*L in *Cryptomonas *(Cryptophyceae)

**DOI:** 10.1186/1471-2148-5-56

**Published:** 2005-10-18

**Authors:** Kerstin Hoef-Emden, Hoang-Dung Tran, Michael Melkonian

**Affiliations:** 1 Universität zu Köln, Botanisches Institut, Lehrstuhl I; Gyrhofstr. 15, 50931 Köln, Germany

## Abstract

**Background:**

Plastid-bearing cryptophytes like *Cryptomonas *contain four genomes in a cell, the nucleus, the nucleomorph, the plastid genome and the mitochondrial genome. Comparative phylogenetic analyses encompassing DNA sequences from three different genomes were performed on nineteen photosynthetic and four colorless *Cryptomonas *strains. Twenty-three *rbc*L genes and fourteen nuclear SSU rDNA sequences were newly sequenced to examine the impact of photosynthesis loss on codon usage in the *rbc*L genes, and to compare the *rbc*L gene phylogeny in terms of tree topology and evolutionary rates with phylogenies inferred from nuclear ribosomal DNA (concatenated SSU rDNA, ITS2 and partial LSU rDNA), and nucleomorph SSU rDNA.

**Results:**

Largely congruent branching patterns and accelerated evolutionary rates were found in nucleomorph SSU rDNA and *rbc*L genes in a clade that consisted of photosynthetic and colorless species suggesting a coevolution of the two genomes. The extremely accelerated rates in the *rbc*L phylogeny correlated with a shift from selection to mutation drift in codon usage of two-fold degenerate NNY codons comprising the amino acids asparagine, aspartate, histidine, phenylalanine, and tyrosine. Cysteine was the sole exception. The shift in codon usage seemed to follow a gradient from early diverging photosynthetic to late diverging photosynthetic or heterotrophic taxa along the branches. In the early branching taxa, codon preferences were changed in one to two amino acids, whereas in the late diverging taxa, including the colorless strains, between four and five amino acids showed changes in codon usage.

**Conclusion:**

Nucleomorph and plastid gene phylogenies indicate that loss of photosynthesis in the colorless *Cryptomonas *strains examined in this study possibly was the result of accelerated evolutionary rates that started already in photosynthetic ancestors. Shifts in codon usage are usually considered to be caused by changes in functional constraints and in gene expression levels. Thus, the increasing influence of mutation drift on codon usage along the clade may indicate gradually relaxed constraints and reduced expression levels on the *rbc*L gene, finally correlating with a loss of photosynthesis in the colorless *Cryptomonas paramaecium *strains.

## Background

Ribulose-1,5-bisphosphate carboxylase/oxygenase (RuBisCO) plays a key role in the photosynthetic Calvin cycle as the carbon dioxide fixating enzyme [1,2]. The most common type of RuBisCO, form I RuBisCO, is found in Viridiplantae, cyanobacteria (green-like RuBisCO), in most non-green algae, and some proteobacteria (red-like RuBisCO) [3]. Eight large subunits and an equal number of small subunits make up a functional holoenzyme of form I RuBisCO [1]. Their genes, *rbc*L and *rbc*S, are plastid-encoded and co-transcribed in non-green algae, whereas in the Viridiplantae, *rbc*S was transferred to the nucleus and evolved to a multi-gene family [3,4]. To compensate for its inefficient and slow catalytic mechanism, RuBisCO is usually expressed at high rates in plastids, making it "the most abundant protein in the world" [5]. Surprisingly, in some colorless algae and holoparasitic land plants, functional RuBisCO was found [6,7]. One possible explanation for a function of RuBisCO outside of the Calvin cycle was reported only recently. In developing *Brassica napus *seeds, RuBisCO recycles CO_2 _that was released in the pyruvate dehydrogenase step prior to fatty-acid biosynthesis [8].

Cryptophyte algae are flagellates with complex plastids originating from a secondary endosymbiosis between a phagotrophic host cell and a red alga [9]. The cryptophyte plastid consists of two nested compartments, each with its own genome (nucleomorph in the periplastidial space between inner and outer pairs of plastid membranes and plastid genome) [9,10]. Cryptophytes of the genus *Cryptomonas *thrive exclusively in freshwater habitats [11]. The leukoplast-bearing freshwater cryptophytes were formerly considered a separate genus *Chilomonas*, but have been shown in phylogenetic analyses to be colorless *Cryptomonas *cells; the diagnosis of the genus *Cryptomonas *was emended accordingly [11]. In previous molecular phylogenetic analyses, accelerated evolutionary rates in either nuclear ribosomal DNA (internal transcribed spacer 2 [ITS2] and partial LSU ribosomal DNA [LSU rDNA]) or nucleomorph SSU ribosomal DNA sequences were found in three independently evolved colorless *Cryptomonas *lineages and in closely related photosynthetic strains [12].

Accelerated evolutionary rates are usually considered indicative for relaxed selective constraints and were also found in *rbc*L genes of land plants [13,14]. Previous studies have shown that relaxed selective constraints and different levels of gene expression in protein-coding genes correlate with biases in codon usage [15,16]. Possibly, by preferring the so-called "major codons" in highly expressed genes, the efficiency of the translation process is increased [17]. The differences in codon usage between highly and lowly expressed genes are most obvious for two-fold degenerate NNY codons, i.e. pairs of triplets that code for the same amino acid with either C or T(U) at third positions. In highly expressed genes, NNC is preferred over NNT in most two-fold degenerate NNY codons, whereas the codon preference reverses, if functional constraints are relaxed and expression levels decrease [16,18]. Under neutral mutation, DNA displays a strong bias towards increased A+T contents. Such a genome compositional bias has been found also in endosymbiotic or organellar genomes [9,19,20]. Thus, in two-fold degenerate NNY codons, codon bias due to selection operates in opposite direction to genome compositional bias [16].

In this study, we compare the phylogeny of the *rbc*L gene as a representative for the plastid genome to phylogenies of nuclear rDNA (concatenated SSU rDNA, ITS2 and partial LSU rDNA) and nucleomorph SSU rDNA. To obtain a congruent taxon sampling across the three genomes of all strains, twenty-three *rbc*L genes and fourteen nuclear SSU rDNA genes were newly sequenced. As putative indicators for differences of functional constraints and expression levels in the *rbc*L genes, codon usages of two-fold degenerate NNY codons were compared among photosynthetic and colorless *Cryptomonas *species.

## Results

### Results of the phylogenetic analyses

**Figure 1:**
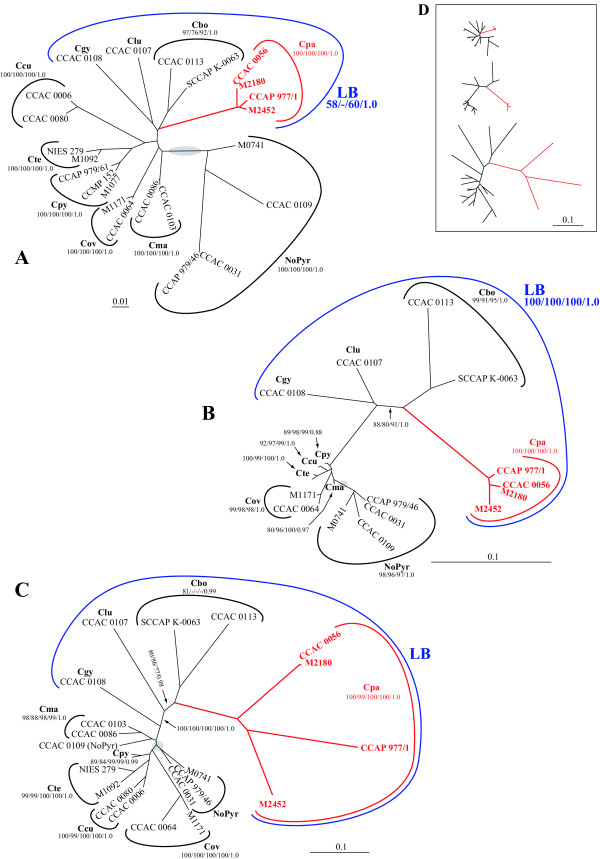
**Unrooted maximum likelihood trees of DNA sequences representing three different genomes of the cryptophyte genus *Cryptomonas***. Figure 1A – Tree inferred from concatenated nuclear SSU rDNA, ITS2 and partial LSU rDNA sequences. Evolutionary model, GTR+I+Γ [51]; -*ln *L = 9254.5. Figure 1B – Nucleomorph SSU rDNA phylogeny. Evolutionary model, TVM+I+Γ [51]; -*ln *L = 4899.1. Figure 1C – Tree inferred from plastid-encoded *rbc*L genes (for a rooted tree including *rbc*L genes of other cryptophyte genera, see Additional file 3). Evolutionary model, GTR+I+Γ [51]; -*ln *L = 7857.4. Figure 1D (inlet) – Nuclear (top), nucleomorph (middle) and plastid (bottom) phylogeny scaled to the same substitution rate. Gray shaded areas in Figures 1A to C, presumed position of the root. In a rooted phylogeny inferred from a concatenated data set of nuclear (ITS2 excluded), nucleomorph and plastid DNA sequences with *Guillardia theta *as an outgroup, the root inserted between clade NoPyr and all other taxa (see Additional file 4). Evolutionary models were chosen according to the results of the Akaike information criterion in Modeltest (see Additional file 1 and Methods). Support values from left to right, maximum likelihood bootstrap/maximum parsimony bootstrap/distance (neighbor-joining) bootstrap/posterior probabilities (Figures 1A and B) or maximum likelihood bootstrap/maximum parsimony bootstrap/distance (neighbor-joining) bootstrap/logdet transformation bootstrap/posterior probabilities (Figure 1C). Cbo, *Cryptomonas borealis*; Ccu, *C. curvata*; Cgy, *C. gyropyrenoidosa*; Clu, *C. lundii*; Cma, *C. marssonii*; Cov, *C. ovata*; Cpa, *C. paramaecium *(colorless); Cpy, *C. pyrenoidifera*; Cte, *C. tetrapyrenoidosa*; blue, taxa of clade LB; red branches and strain designations, loss of photosynthesis; scale bars, substitutions per site.

Both ribosomal data sets passed the chi-square test for homogeneity of base frequencies across taxa, whereas the *rbc*L data set failed the test (Additional file 1). After exclusion of third codon positions, the *rbc*L data set passed the test, indicating that the heterogeneity of base frequencies was restricted to third codon positions. This became also obvious by separate computation of mean values and standard deviations of base frequencies for first, second and third codon positions across all twenty-three taxa. The second codon position was most homogeneous concerning standard deviations (A: 27.8 ± 0.3%; C: 21.4 ± 0.4%; G: 19.9 ± 0.4%; T: 30.9 ± 0.3%), the first codon position had an intermediate position (A: 24.6 ± 1.0%; C: 17.8 ± 1.4, G: 38.9 ± 0.7, T: 18.7 ± 1.1), whereas the third codon position was most heterogeneous (A: 29.0 ± 2.6%; C: 16.1 ± 4.6%; G: 10.3 ± 3.8%; T: 44.6 ± 3.2%). Phylogenetic analyses with bootstrap resampling under all optimality criteria and a Bayesian analysis, however, showed that despite of an obvious bias, these positions contributed the most to the support of the clades in the *rbc*L data set. This was confirmed by separate phylogenetic analyses of first, second and third codon positions (Additional file 1). The highly conserved *rbc*L protein sequences consistently failed to recover three clades that were otherwise highly supported in all DNA sequence data sets (*Cryptomonas marssonii*, *C. ovata *and *C. pyrenoidifera*), because phylogenetic information was predominantly based on synonymous substitutions. Therefore, we used the *rbc*L gene tree with complete codon positions for a comparison with the nuclear and nucleomorph ribosomal DNA phylogenies (Figure 1A to 1C).

Almost all *Cryptomonas *clades were unequivocally recovered with significant or at least moderate support in nuclear, nucleomorph and plastid gene trees (Figure 1A to 1C). This refers to *C. curvata *(Ccu), *C. marssonii *(Cma), *C. ovata *(Cov), *C. paramaecium *(colorless strains; Cpa), *C. pyrenoidifera *(Cpy), and *C. tetrapyrenoidosa *(Cte; clades named according to Hoef-Emden and Melkonian 2003). *C. borealis *(Cbo) was significantly supported in nuclear and nucleomorph phylogenies but not in all phylogenetic analyses in the *rbc*L phylogeny (Figure 1C). Significant support for this clade, however, was found in the *rbc*L protein phylogeny apparently due to nonsynonymous substitutions (tree not shown; for support values, see Additional file 1). Clade NoPyr (for no pyrenoids [11]), otherwise highly supported in nuclear and nucleomorph phylogenies, could not be resolved in the *rbc*L phylogeny (Figure 1C).

In all phylogenies, *C. borealis*, *C. gyropyrenoidosa *and *C. lundii *formed a "super-clade" together with the colorless *C. paramaecium *(termed clade LB for long-branch in [11]; Figure 1A to 1C). Only in the nucleomorph SSU rDNA tree, however, convincing support for clade LB was found (Figure 1B). In the nucleomorph SSU rDNA and *rbc*L phylogenies, representing the two genomes of the complex plastid, evolutionary rates and topologies of the strains in clade LB resembled each other. In both phylogenies, evolutionary rates were extremely accelerated in clade LB, and the branching pattern was similar, except for the position of *C. gyropyrenoidosa*, which was a sister to *C. lundii *in the nucleomorph SSU rDNA (but without bootstrap support), but not in the *rbc*L phylogeny (where it was the first divergence). In the nuclear-encoded ribosomal DNA phylogeny, predominantly the strains of clade NoPyr displayed increased evolutionary rates, whereas evolutionary rates were less pronounced in *C. borealis *and *C. paramaecium *of clade LB (Figure 1A). In clade NoPyr, an acceleration of evolutionary rates was also present to a lesser extent in the nucleomorph SSU rDNA; in the *rbc*L phylogeny, however, branch lengths of this clade were inconspicuous (Figure 1B and 1C).

**Figure 2:**
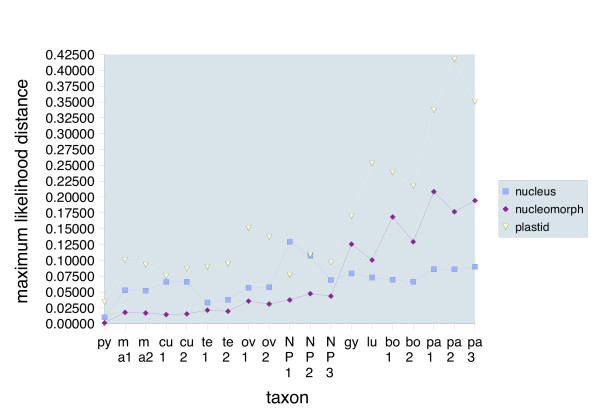
**Chart diagram displaying genetic divergences among the taxa and across the three data sets**. A strain from a clade with inconspicuous branch lengths in all three phylogenies, *Cryptomonas pyrenoidifera *strain M1077, was chosen as a reference. The distance values represent the genetic divergences of strain M1077 to the other taxa. The distance values were extracted from the maximum likelihood distance matrices used otherwise by Paup to infer the neighbor-joining trees during phylogenetic analyses, and fed into a spread-sheet program. Strains CCMP 152, CCAC 0031 and M2180 were genetically identical to strains M1077, CCAP 979/46 and CCAC 0056, respectively, thus, were omitted from the chart diagram. Nucleus, concatenated nuclear SSU rDNA, ITS2 and partial LSU rDNA; nucleomorph, nucleomorph SSU rDNA; plastid, *rbc*L gene. Taxon designations (abscissa): py, *C. pyrenoidifera *CCAP 979/61; ma1, *C. marssonii *CCAC 0086; ma2, *C. marssonii *CCAC 0103; cu1, *C. curvata *CCAC 0006; cu2, *C. curvata *CCAC 0080; te1, *C. tetrapyrenoidosa *M1092; te2, *C. tetrapyrenoidosa *NIES 279; ov1, *C. ovata *CCAC 0064; ov2, *C. ovata *M1171; NP1, NoPyr strain CCAP 979/46; NP2, NoPyr strain CCAC 0109; NP3, NoPyr strain M0741; gy, *C. gyropyrenoidosa *CCAC 0108; lu, *C. lundii *CCAC 0107; bo1, *C. borealis *CCAC 0113; bo2, *C. borealis *SCCAP K-0063; pa1, *C. paramaecium *M2452; pa2, *C. paramaecium *CCAP 977/1; pa3, *C. paramaecium *CCAC 0056.

In Figure 1D, the phylogenetic trees of Figure 1A to 1C were scaled to the same substitution rate and in Figure 2, the maximum likelihood distances of *C. pyenoidifera *strain M1077 to the other taxa were plotted in a chart diagram for a direct comparison of genetic divergences. Among the three data sets, the *rbc*L data displayed generally the highest substitution rates and genetic distances. In the nucleomorph SSU rDNA, the evolutionary rates of *C. gyropyrenoidosa*, *C. borealis *and *C. paramaecium *were in an intermediate position. Apparently, evolutionary rates in clade LB increased successively from host to nucleomorph to plastid genome.

### Codon usage analysis

In Table 1, the codon usages of the six amino acids with two-fold degenerate NNY codons (asparagine, histidine, aspartate, tyrosine, cysteine and phenylalanine) are listed in absolute counts computed from the 396 codons that were included in the phylogenetic analyses. Cysteine was exceptional in codon usage in that it mostly showed a preference for UGU over UGC, thus it will not be further discussed (Table 1). For the remaining five amino acids, NNC codons were always preferred over NNU codons in *C. marssonii*, *C. pyrenoidifera*, *C. tetrapyrenoidosa *and in clade NoPyr (Table 1). In all strains of clade LB, on the other hand, indications for a change of codon usage were found, although to different extent. In almost all strains of *C. paramaecium *(except for histidine in strain CCAP 977/1) and *C. borealis *(except for asparagine in strain SCCAP K-0063) codon preferences were inversed from NNC to NNU for these amino acids (Table 1). *C. lundii *and *C. gyropyrenoidosa *were in an intermediate position concerning codon usages. In *C. lundii *only in two amino acids, aspartate and tyrosine, codon usage was inversed to prefer GAU over GAC (aspartate) and UAU over UAC (tyrosine), whereas in *C. gyropyrenoidosa *only one amino acid, aspartate, was affected (Table 1). Inversed codon usages were also found in two clades that were not part of LB, *C. curvata *(histidine and aspartate in strain CCAC 0080, aspartate in strain CCAC 0006) and *C. ovata *(aspartate and tyrosine; Table 1). In the three phylogenies, *C. ovata *displayed slightly increased evolutionary rates in nucleomorph SSU rDNA and *rbc*L phylogenies, whereas *C. curvata *had slightly longer branches only in the nuclear ribosomal DNA phylogeny (Figure 1A to 1C).

**Table 1:** Codon usage of two-fold degenerate NNY codons in *Cryptomonas *sp. and *Guillardia theta rbc*L

Clade	Asn	His	Asp	Tyr	Cys	Phe
Strain	AAC/AAU	CAC/CAU	GAC/GAU	UAC/UAU	UGC/UGU	UUC/UUU
*Guillardia theta*	15/0	8/2	12/8	12/3	0/7	12/5
*C. curvata*						
CCAC 0006	13/2	7/3	**7/13**	10/5	4/5	14/3
CCAC 0080	13/2	**4/6**	10/10	11/4	3/6	11/6
*C. marssonii*						
CCAC 0086	16/0	6/4	14/6	12/3	1/7	11/6
CCAC 0103	15/1	6/4	10/9	13/2	2/5	10/3
*C. ovata*						
CCAC 0064	8/8	8/2	**7/13**	**5/10**	0/8	10/7
M1171	10/6	5/5	**7/13**	**5/10**	1/7	10/7
*C. pyrenoidifera*						
CCAP 979/61	15/0	8/2	12/8	13/3	0/7	12/5
CCMP 152 and M1077	15/0	9/1	13/7	12/3	1/6	13/4
*C. tetrapyrenoidosa*						
M1092	13/3	8/2	13/6	10/5	3/5	12/5
NIES 279	12/4	8/2	10/9	10/5	1/7	13/4
NoPyr						
CCAC 0031 and CCAP 979/46	15/0	10/0	14/6	13/2	4/3	16/1
CCAC 0109	15/0	9/1	15/5	10/5	1/5	15/2
M0741	15/0	6/4	13/7	9/6	3/3	15/2
clade LB (basally diverging taxa first)						
*C. gyropyrenoidosa*						
CCAC 0108	12/4	5/5	**9/10**	11/4	1/5	12/6
*C. lundii*						
CCAC 0107	12/4	6/4	**4/15**	**4/11**	0/7	9/8
*C. borealis*						
CCAC 0113	**6/9**	**2/8**	**2/18**	**3/12**	0/8	**5/12**
SCCAP K-0063	8/7	**2/8**	**5/14**	**4/11**	1/7	**5/12**
*C. paramaecium*						
CCAC 0056 and M2180	**6/9**	**3/7**	**5/15**	**6/9**	0/7	**4/13**
CCAP 977/1	**6/9**	6/4	**4/16**	**4/11**	1/6	**5/12**
M2452	**5/10**	**3/7**	**7/12**	**7/8**	1/6	**6/11**

The entries for the *rbc*L genes in Table 1 refer to absolute counts per 396 amino acids (= 1188 nucleotide positions). Also for the *Guillardia theta rbc*L gene only the corresponding 396 of the 488 codons were used. The plastid of *G. theta *contains genes for 30 tRNAs [10]. Only the NNC codons (underlined) are served by an exactly matching tRNA. As a code table, the eubacterial/plastid code was used (code table 11). Bold face, changed codon usage from NNC to NNU = shift from selection bias to genome composition bias.

## Discussion

### Lineage-specific parallel evolution across three genomes in *Cryptomonas*

Most of the *Cryptomonas *clades were recovered with high support values in phylogenies of the concatenated nuclear ribosomal DNA sequences (SSU rDNA, ITS2 and partial LSU rDNA), of the nucleomorph SSU rDNA and of the plastid-encoded *rbc*L gene, but obvious differences in evolutionary rates among the different clades and genomes were displayed.

In the "super-clade" LB, consisting of three photosynthetic (*C. borealis*, *C. gyropyrenoidosa *and *C. lundii*) and one heterotrophic *Cryptomonas *species (*C. paramaecium*), largely congruent branching patterns and extreme evolutionary rates in the nucleomorph and plastid gene phylogenies suggested coevolution under similar selective constraints, as if the two genomes of the complex plastid were a genetic unit in this clade. In the nuclear ribosomal DNA phylogeny, an increase in evolutionary rates was in part also present but less pronounced. Support for this clade was low in the nuclear ribosomal DNA phylogeny, although several parts of the nuclear ribosomal operon were concatenated to improve resolution (the nuclear SSU rDNA alone failed to recover clade LB, but increased evolutionary rates in *C. paramaecium *and *C. borealis *were more pronounced than in the concatenated data set; not shown).

In a different clade, that also consists of photosynthetic and colorless *Cryptomonas *taxa, clade NoPyr, the situation was reversed; coevolution with increased evolutionary rates seemed to have taken place in the nuclear and nucleomorph genes [12], whereas no acceleration of evolutionary rates could be observed in the *rbc*L gene phylogeny (this study). We did not obtain an *rbc*L PCR product, however, from the colorless strains of clade NoPyr [[12], this study].

Cho et al. [21] demonstrated that extremely accelerated evolutionary rates were present in three mitochondrial genes (two protein-coding genes, *cox*1, *atp*1, and one RNA-coding gene, *rrn*16, the gene for the SSU rDNA in mitochondria) in the flowering plant genus *Plantago*, but not in plastid or nuclear genes of the same taxa. We chose two RNA-coding genes and a protein-coding gene as representatives for three of the four genomes in *Cryptomonas*. Despite their differing functions, the phylogenetic trees suggested that at least two (clade NoPyr), or even all three genomes (clade *C. borealis *and *C. paramaecium*) may have evolved in parallel under similar selective constraints or by interacting with each other.

### Evidence for relaxed functional constraints on RNA- or protein-coding genes

Possible explanations for accelerated evolutionary rates of DNA sequences include relaxation or loss of functional constraints due to either changes in mode of nutrition, adaptations to new environmental conditions, genetic bottlenecks or obligate asexuality [22-25]. Endosymbiotic, parasitic and organellar genomes are notorious for high A+T contents in their genomes likely caused by biased substitution rates under neutral mutational pressure [19,20,26,27]. Minimum amounts of G and C are required to maintain the codon information for a functional protein or to preserve the secondary structure of an RNA. Depending on the strengths of the functional constraints, the resulting selection bias may differ from the genome composition bias to varying degrees (reviewed for protein-coding genes in [16]). Thus, lineage-specific relaxed selective constraints may be identified by increases in A+T content.

This notion is supported by the observation that the nucleomorph SSU rRNA genes in clade LB accumulate mononucleotide repeats of A and T in highly variable regions [11,12]. In functional protein-coding genes, the triplet structure constrains mutation rates by selection. Synonymous substitutions do not replace amino acids, thus are more likely to occur than nonsynonymous substitutions. In previous studies, however, also synonymous substitutions were reported to be skewed towards specific codons in correlation with expression levels of the respective protein [15,28,29]. The codon biases were explained as a result of a competition between selection and genome compositional bias [17]. In highly expressed genes, codons with abundant or perfectly matching tRNAs (major codons) are apparently preferred over codons are translated by rare or "wobbling" tRNAs (minor codons) [29,30]. In plastid genomes, usually only 30 to 31 tRNAs are available to translate all 61 codons (in the *Guillardia theta *plastome, 30 tRNAs were found) [10,16]. In two-fold degenerate NNY codons, the preferred major codon in highly expressed genes is usually NNC, thus codon bias due to selection can be comparably easily distinguished from codon bias due to mutation drift [16,18]. Among the six amino acids with two-fold degenerate codons (asparagine, aspartate, histidine, tyrosine, phenylalanine and cysteine), cysteine seems to be the sole exception [[16], this study].

In the *rbc*L genes of most *Cryptomonas *clades, the major NNC codons for asparagine, aspartate, histidine, tyrosine, or phenylalanine were preferred over their NNU alternatives, however, codon preferences were reversed in several or all of these amino acids in clade LB [this study]. There was even a gradient of decreasing selective constraints and presumably also expression levels along the LB clade: In the early diverging *C. gyropyrenoidosa *and *C. lundii*, reversed codon usages were found in only one or two amino acids, whereas in the late diverging *C. borealis *and *C. paramaecium *in four or five NNY-coded amino acids, NNU codons were preferred. Morton and Levin used the codon adaptation index (CAI) to compare codon usage of two-fold degenerate NNY codons in *psb*A genes among dicot and monocot plants, and discussed putatively decreasing selective constraints from basally to terminally diverging lineages [18]. However, no hemi- or holoparasitic angiosperm plants were included in their study.

In previous studies, convergent codon usage resulted in artificial tree topologies [31]. Despite an obvious bias in codon usage, the *rbc*L phylogeny was largely confirmed by the nuclear and nucleomorph ribosomal DNA phylogenies. It is likely that the *rbc*L genes examined in this study had not yet diverged enough to cause artifacts. It may have been different, though, if *rbc*L had been used to infer phylogenetic trees across cryptophyte genera. For higher level phylogenies, it may, thus, be a better option to use protein sequences instead.

### Potential causes for lineage-specific accelerated rates and relaxed functional constraints

One of the explanations for accelerated evolutionary rates and relaxed functional constraints in plastid genomes is loss of photosynthesis, since this usually results in large-scale degradation and compaction of plastomes leading to loss of almost all photosynthetic genes, except perhaps for *rbc*L [22,32,33].

In the cryptophyte *Guillardia theta*, photosynthesis genes are spread across plastid (46 genes), nucleomorph (30 genes) and nucleus (α-subunits of phycoerythrin, and an unknown number of additional photosynthetic genes) [9,10,34,35]. Thus, loss of photosynthesis is not an unlikely explanation for a parallel acceleration of evolutionary rates across three genomes in *C. paramaecium*. However, observations of elevated evolutionary rates in closely related photosynthetic taxa contradict this notion. Instead of being the cause for increased substitution rates, loss of photosynthesis may rather be a result of an accelerated evolution that had started already in the photosynthetic ancestors of the colorless lineages [[12], this study]. Similar observations have been made in plastid gene phylogenies of hemi- and holoparasitic land plants [36].

Previous studies have shown that mutations in genes of DNA repair or DNA replication may result in overall increases of substitution rates in bacterial and eukaryotic genomes, e.g. [37]. Many genes of the cryptophyte nucleomorph have been transferred to the host nucleus including DNA polymerases [9,10,35]. Thus, some potential mutator genes in cryptophytes can be expected to be nuclear-encoded. It is tempting to speculate that a spontaneous mutation in a nuclear-encoded plastid-targeted protein, for example in the proofreading subunit of a DNA polymerase III or in a DNA repair enzyme, could have accelerated successively mutation rates in nuclear, nucleomorph and plastid DNA in the photosynthetic ancestors of clade LB. Accelerated mutation rates may have resulted in loss of photosynthesis in *C. paramaecium*, which in turn perhaps resulted in less functional constraints on the *rbc*L protein and, thus, in further increase of evolutionary rates.

Another possible cause for accelerated evolutionary rates in clade LB was discussed previously [12]. The genus *Cryptomonas *is dimorphic, a feature that usually correlates with sexual reproduction. Final proof for sexual reproduction is still missing, but, however, in clade LB only strains with campylomorph cells were found[11,12]. It may, thus, as well be possible that loss of sexual reproduction caused an increase in mutation rates by loss of recombination affecting also nuclear-encoded plastid-targeted proteins. However, the observed increase in evolutionary rates from host to nucleomorph to plastid genome suggests that the evolutionary processes may have started in the plastid genome.

## Conclusion

An *rbc*L phylogeny of twenty-three *Cryptomonas *strains was compared with phylogenetic trees inferred from nucleomorph and nuclear ribosomal DNA sequences. In a super-clade comprising photosynthetic and colorless *Cryptomonas *species, a congruent increase in evolutionary rates and a similar branching pattern were found in data sets representing the two genomes of the complex plastid, the nucleomorph SSU rDNA and the *rbc*L data set. In both data sets, the colorless strains displayed the highest substitution rates. A direct comparison of the genetic distances across nuclear, nucleomorph and plastid data sets showed that the evolutionary rates in the long-branch super-clade were highest in the *rbc*L genes and lowest in the nuclear ribosomal DNA. Perhaps evolutionary rates first accelerated in the plastid genome, then in the nucleomorph genome. The increased evolutionary rates of nucleomorph SSU rDNA and *rbc*L gene evolved in parallel with a gradual shift in codon usage of the *rbc*L gene towards a relax in functional constraints and decreasing expression levels. Strongest evidence for relaxed functional constraints and decreased expression levels in *rbc*L were found in the terminally diverging photosynthetic species *Cryptomonas borealis *and in the colorless species *C. paramaecium*. Either loss of photosynthesis was a gradual at first hidden process starting already in pigmented ancestors of the colorless *C. paramaecium *strains or the accelerated evolutionary rates caused defects in the photosynthetic genes resulting in loss of photosynthesis.

## Methods

### Algal cultures

Photosynthetic and heterotrophic *Cryptomonas *strains were obtained from different algal culture collections (Table 2). Photosynthetic strains were maintained in modified WARIS-H freshwater culture medium [38,39], and heterotrophic strains in biphasic soil/water medium with one-eighth of a pea for supply with organic substances. Strains were grown at 15°C under a 14/10 h light/dark regime (15–35 μmol photons m^-2 ^s^-1^; photosynthetic strains) or in the dark (colorless strains).

**Table 2:** List of *Cryptomonas *strains examined in this study with accession numbers to EMBL/GenBank/DDBJ entries

Species/Clade	Strain	Nucleus		Nucleom.	Plastid
		ITS2+LSUp	SSU rDNA	SSU rDNA	*rbc*L
*C. borealis*	CCAC 0113	[EMBL:AJ566160]	[EMBL:AM051188]	[EMBL:AJ566185]	[EMBL:AM051202]
	SCCAP K-0063	[EMBL:AJ566159]	[EMBL:AJ420696]	[EMBL:AJ420688]	[EMBL:AM051203]
*C. curvata*	CCAC 0006	[EMBL:AJ566147]	[EMBL:AJ007280]	[EMBL:AJ420682]	[EMBL:AM051204]
	CCAC 0080	[EMBL:AJ566148]	[EMBL:AM051189]	[EMBL:AJ715462]	[EMBL:AM051205]
*C. gyropyrenoidosa*	CCAC 0108	[EMBL:AJ566154]	[EMBL:AJ421149]	[EMBL:AJ420686]	[EMBL:AM051206]
*C. lundii*	CCAC 0107	[EMBL:AJ566161]	[EMBL:AM051190]	[EMBL:AJ566184]	[EMBL:AM051207]
*C. marssonii*	CCAC 0086	[EMBL:AJ566155]	[EMBL:AM051191]	[EMBL:AJ566173]	[EMBL:AM051208]
	CCAC 0103	[EMBL:AJ715444]	[EMBL:AM051192]	[EMBL:AJ566174]	[EMBL:AM051209]
*C. ovata*	CCAC 0064	[EMBL:AJ566153]	[EMBL:AM051193]	[EMBL:AJ566178]	[EMBL:AM051210]
	M1171	[EMBL:AJ566152]	[EMBL:AJ420695]	[EMBL:AJ420687]	[EMBL:AM051211]
*C. paramaecium*	CCAC 0056	[EMBL:AJ566158]	[EMBL:AJ007276]	[EMBL:AJ420676]	[EMBL:AM051212]
	CCAP 977/1	[EMBL:AJ715445]	[EMBL:AM051194]	[EMBL:AJ715465]	[EMBL:AM051213]
	M2180	[EMBL:AJ715451]	[EMBL:AM051195]	[EMBL:AJ715471]	[EMBL:AM051214]
	M2452	[EMBL:AJ715452]	[EMBL:AM051196]	[EMBL:AJ715472]	[EMBL:AM051215]
*C. pyrenoidifera*	CCAP 979/61	[EMBL:AJ566142]	[EMBL:AJ421147]	[EMBL:AJ420684]	[EMBL:AM051216]
	CCMP 152	[EMBL:AJ566140]	[EMBL:AJ421150]	[EMBL:AJ420675]	[EMBL:AM051217]
	M1077	[EMBL:AJ566144]	[EMBL:AM051197]	[EMBL:AJ566180]	[EMBL:AM051218]
*C. tetrapyrenoidosa*	M1092	[EMBL:AJ566146]	[EMBL:AM051198]	[EMBL:AJ566182]	[EMBL:AM051219]
	NIES 279	[EMBL:AJ715455]	[EMBL:AM051199]	[EMBL:AJ566183]	[EMBL:AM051220]
NoPyr	CCAC 0031	[EMBL:AJ566166]	[EMBL:AJ007281]	[EMBL:AJ420685]	[EMBL:AM051221]
	CCAC 0109	[EMBL:AJ566165]	[EMBL:AJ420697]	[EMBL:AJ420683]	[EMBL:AM051222]
	CCAP 979/46	[EMBL:AJ566167]	[EMBL:AM051200]	[EMBL:AJ566171]	[EMBL:AM051223]
	M0741	[EMBL:AJ566163]	[EMBL:AM051201]	[EMBL:AJ566172]	[EMBL:AM051224]

Abbreviations: CCAC, Culture Collection of Algae at the University of Cologne (Germany); CCAP, Culture Collection of Algae and Protozoa (UK); CCMP, Provasoli-Guillard Center for the Culture of Marine Phytoplankton (USA); ITS2, nuclear internal transcribed spacer 2; LSUp, partial nuclear large subunit ribosomal DNA (28S rDNA or LSU rDNA, approx. 800 nt of 5' terminus); M, Algal Culture Collection Melkonian at the University of Cologne (Germany); NIES, Culture Collection of The National Institute for Environmental Studies (Japan); *rbc*L, large subunit gene of ribulose-1,5-bisphosphate carboxylase/oxygenase; SCCAP, Scandinavian Culture Centre for Algae and Protozoa (Denmark); SSU rDNA, small subunit ribosomal DNA (18S rDNA, nuclear or nucleomorph).

New sequences: acc. nos. AM051188 to AM051224.

### Isolation of DNA, PCR amplification and sequencing

Total genomic DNA was isolated from the cells with the DNeasy Plant Mini Kit according to the manufacturer's protocol (Qiagen, Hilden, Germany). PCR amplification of nuclear SSU rDNA, ITS2 and partial LSU rDNA, and of nucleomorph SSU rDNA with nucleus- or nucleomorph-specific primers followed previously described protocols [11,40]. For PCR amplification of cryptophyte *rbc*L genes, new primers were designed using an alignment of bangiophyte or florideophycean red algal and cryptophyte *rbc*L sequences (cryptophyte sequences: *Chroomonas *sp., acc. no. AY119781; *Cryptomonas paramaecium*, acc. no. AY119780; *Guillardia theta*, acc. no. AF041468; *Pyrenomonas helgolandii*, acc. no. AY199782). Similarly, new sequencing primers were constructed using the same alignment (sequences of PCR primers and sequencing primers for *rbc*L are listed in Additional file 2). For PCR amplification of *rbc*L DNA sequences, the same cycling protocol as for the ribosomal DNA sequences was used except for a decrease of the annealing temperature (predenaturation for 3 min. at 95°C; 30 cycles: 1 min. at 95°C, 2 min. at 45 or 50°C, 3 min. at 68°C). PCR products were purified with the Dynabead M-280 system according to the manufacturer's protocol (Dynal, Oslo, Norway). For bidirectional sequencing, two sets of primer pairs were used for each PCR product; the forward primers were labeled with IRDye-800 and the reverse primers with IRDye-700 (see Additional file 2). Double-stranded sequences were determined with a Li-Cor 4200L bidirectional sequencer (Li-Cor Biosciences, Bad Homburg, Germany).

### Phylogenetic analyses

The *rbc*L nucleotide and protein sequences were prealigned with clustalw and refined by eye using the multiple alignment sequence editor SeaView [41]. The ribosomal DNA sequences were manually aligned according to secondary structure; non-alignable regions were excluded prior to the phylogenetic analyses.

Since the taxon sampling was congruent for plastid-, nucleomorph- and nucleus-encoded sequences, all unrooted data sets comprised 23 taxa (accession nos. are listed in Table 2). The unrooted *rbc*L nucleotide data set consisted of 1188 positions and was translated to perform phylogenetic analyses of protein sequences or modified for phylogenetic analyses of single codon positions (396 positions each data set; see Additional file 1 for additional information about the data sets). A rooted data set of *rbc*L nucleotide sequences consisted of 46 taxa and 990 positions, including 14 rhodophyte *rbc*L sequences as outgroup taxa (Additional file 3). The nuclear ribosomal DNA sequences were concatenated for phylogenetic analyses resulting in a data set with a total length of 2623 nucleotides (complete nuclear ITS2 and partial nuclear LSU rDNA comprising approx. 800 nt of the 5' terminus: 1083 positions; nuclear SSU rDNA: 1540 positions). The nucleomorph SSU rDNA data set comprised 1496 positions.

All nucleotide data sets were subjected to distance, maximum likelihood, maximum parsimony and Bayesian analyses. To determine the evolutionary model fitting best the data according to the Akaike Information Criterion (AIC), Modeltest 3.6 was used [42]). Distance, maximum likelihood and maximum parsimony analyses were performed with the program PAUP* 4.0b10 [43]. Distance analyses were run under minimum evolution and set to the maximum likelihood parameters proposed by Modeltest. Data sets with heterogeneous base frequencies were also analyzed using the LogDet transformation. For both types of analyses, trees were inferred with the neighbor-joining algorithm. Maximum likelihood analyses were done using the proposed evolutionary model settings of Modeltest with three random addition replicates and heuristic tree search algorithm with tree bisection and reconnection (TBR). Unweighted maximum parsimony analyses were performed using 10 random addition replicates also in combination with the heuristic tree search algorithm. For all analyses under the distance or maximum parsimony criterion, 1000 bootstrap replicates were calculated; for maximum likelihood, 500 bootstrap replicates were computed. Bayesian analyses were performed using MrBayes 3.0B4 [44]. For the nucleotide data sets, likelihood settings were set to GTR, gamma-distributed among-site rate variation and covarion (includes proportion of invariable sites). Samples were drawn every 100^th ^generation for at least 3.5 million generations with one cold and three heated chains. Burn-in was determined for the individual data set according to the sump plot.

The protein data set was also subjected to distance, maximum likelihood, maximum parsimony and Bayesian analyses. The evolutionary model fitting best the data was determined with ProtTest 1.2.6 according to the AIC [45,46] and used for maximum likelihood analysis with Phyml 2.4.4 [47]. Distance analysis was performed using protdist from the Phylip 3.62 package (set to JTT+Γ with global rearrangements; progam suite by Joe Felsenstein [48]). The shape parameter α for the gamma distribution in protdist was calculated using Tree-Puzzle 5.2 [49]. Maximum parsimony analysis was done using PAUP* 4.0b10 (10 random addition sequence replicates). For Bayesian analyses with MrBayes 3.0b4, prior expectations were set to AAmodel=mixed (priors for all amino acid substitution matrices considered equal) and likelihood settings to gamma-distributed among-site rate variation and proportion of invariable sites. Samples were drawn every 100^th ^generation for 5 million generations using one cold and three heated chains. Burn-in was determined according to the sump plot.

### Codon usage analysis

The countcodon program from the web site of the Codon Usage Database [50] was used to determine absolute counts of all codons of the twenty-three *rbc*L sequences (396 codons). The DNA sequences were translated using the eubacterial/plastid codon table (code table 11).

## Authors' contributions

KHE sequenced fourteen nuclear SSU rDNAs, five *rbcL *sequences, aligned the nuclear and nucleomorph data sets, performed the phylogenetic analyses and the codon usage analysis, and wrote the manuscript; HDT sequenced eighteen *rbcL *sequences and did the *rbc*L alignment; MM contributed to planning of the study and critically revised the manuscript. All authors read and approved the final manuscript.

## Supplementary Material

Additional File 1 Evolutionary models and support values for the *Cryptomonas *clades in the unrooted *rbc *L data set. Click here for file

Additional File 2 PCR and sequencing primers for cryptophyte *rbc*L Click here for file

Additional File 3 Rooted maximum likelihood tree of cryptophyte *rbc *L sequences including all codon positions. Click here for file

Additional File 4 Rooted maximum likelihood tree of concatenated nuclear SSU rDNA, partial nuclear LSU rDNA, nucleomorph SSU rDNA and *rbc *L sequences Click here for file
