# Supplementary material for: Lineage-specific variations of congruent evolution among DNA sequences from three genomes, and relaxed selective constraints on rbcL in Cryptomonas (Cryptophyceae)
Source: BMC Evol Biol. 2005 Oct 18;5:56. doi: 10.1186/1471-2148-5-56 (PMC1285359; doi:10.1186/1471-2148-5-56)
Supplement: Additional File 1 — Evolutionary models and support values for the Cryptomonas clades in the unrooted rbc L data set. [file 1471-2148-5-56-S1.pdf]

Additional file 1 – Evolutionary models and support values for the *Cryptomonas* clades in the unrooted rbcL data set

	All Codon Positions	Position 1	Position 2	Position 3	Protein
ntax	23	23	23	23	23
npos	1188	396	396	396	369
basefreq (P)	**0.0163**	1.000	1.000	**0.000**	passed
evol. model	GTR+I+Γ	GTR+I+Γ	TVM+I	GTR+I+Γ	CpRev+I+Γ
Clades	Support				
	ML/MP/NJ/LD/PP	ML/MP/NJ/PP	ML/MP/NJ/PP	ML/MP/NJ/LD/PP	ML/MP/NJ/PP
C. borealis	81/-/-/-/0.99	60/+/+/0.76	-/-/-/0.60	+/-/-/-/53	83/80/90/1.00
C. curvata	100/99/100/100/1.00	60/+/55/0.97	54/59/70/0.94	99/99/99/93/1.00	97/75/97/0.89
C. marssonii	98/88/98/99/1.00	+/-/(+)/-	-/-/+/0.57	69/76/73/72/0.90	-
C. ovata	100/100/100/100/1.00	51/+/63/0.55	-	98/99/99/99/1.00	-/+/+/-
C. paramaecium	100/100/100/99/1.00	99/97/99/1.00	62/71/75/1.00	99/98/99/80/1.00	98/98/99/1.00
C. pyrenoidifera	89/84/99/99/0.99	-	-	87/85/97/87/1.00	+/-/-/0.61
C. pyrenoidosa without CCAP 979/61	97/100/100/100/1.00	+/76/94/0.84	-/+/58/-	98/100/99/99/1.00	97/62/94/0.98
C. tetrapyrenoidosa	99/99/100/100/1.00	88/83/90/0.99	59/59/70/0.96	84/90/97/87/0.99	96/94/94/1.00

Clade designations according to [11]. Clades LB and NoPyr were not supported in the *rbc*L phylogeny. Basefreq (P), probability for a homogeneity of base frequencies or amino acids across taxa (for DNA determined with PAUP*; for amino acids determined with Tree-Puzzle; red, rejected, i.e. base frequencies were heterogenous); CpRev, general time reversible model for plastid-encoded genes [52]; evol. model, evolutionary model according to the Akaike Information Criterion; Γ, gamma-distributed among-site rate variation; GTR, the general time reversible model [51]; I, proportion of invariable sites; LD, logdet/neighbor-joining bootstrap; ML, maximum likelihood bootstrap; MP, maximum parsimony bootstrap; NJ, distance/neighbor-joining bootstrap; npos, number of positions in the data set; ntax, number of taxa in the data set; PP, posterior probability (Bayesian analysis); TVM, the transversion model [51]; +, present but not supported; - , not present
