# Supplementary material for: Lineage-specific variations of congruent evolution among DNA sequences from three genomes, and relaxed selective constraints on rbcL in Cryptomonas (Cryptophyceae)
Source: BMC Evol Biol. 2005 Oct 18;5:56. doi: 10.1186/1471-2148-5-56 (PMC1285359; doi:10.1186/1471-2148-5-56)
Supplement: Additional File 2 — PCR and sequencing primers for cryptophyte rbcL [file 1471-2148-5-56-S2.pdf]

Additional file 2 - PCR and sequencing primers for cryptophyte *rbc*L

Primer	Sequence (5' to 3')
PCR primers	
rbcL1F	CAA GGA GGA AWA YAT GTC TCA ATC
rbcL2F	AGG AGG AAW AYA TGT CTC AAT CCG
rbcL1R$_{biot.}$	TCA GCT GTA TCW GTA GAA GC
Sequencing primers	
rbcL2F-800	AGG AGG AAW AYA TGT CTC AAT CCG
rbcL728F-800	CTC CAR CCW TTY ATG AGA TGG
rbcL1R-700	TCA GCT GTA TCW GTA GAA GC
rbcL1046R-700	ACC WGC CAT RCG CAT CCA CTT AC

The PCR primer rbcL2F was used for semi-nested reamplification. Primers rbcL2F-800 and rbcL1046R-700 or rbcL728F-800 and rbcL1R-700 were combined for bidirectional sequencing and cover the first two thirds or the last two thirds of the gene, respectively. The reverse PCR primer was 5' biotinylated. F, forward primer; R, reverse primer; 800, labeled with IRDye-800; 700, labeled with IRDye-700.
