# Supplementary material for: Lineage-specific variations of congruent evolution among DNA sequences from three genomes, and relaxed selective constraints on rbcL in Cryptomonas (Cryptophyceae)
Source: BMC Evol Biol. 2005 Oct 18;5:56. doi: 10.1186/1471-2148-5-56 (PMC1285359; doi:10.1186/1471-2148-5-56)
Supplement: Additional File 3 — Rooted maximum likelihood tree of cryptophyte rbc L sequences including all codon positions. [file 1471-2148-5-56-S3.pdf]

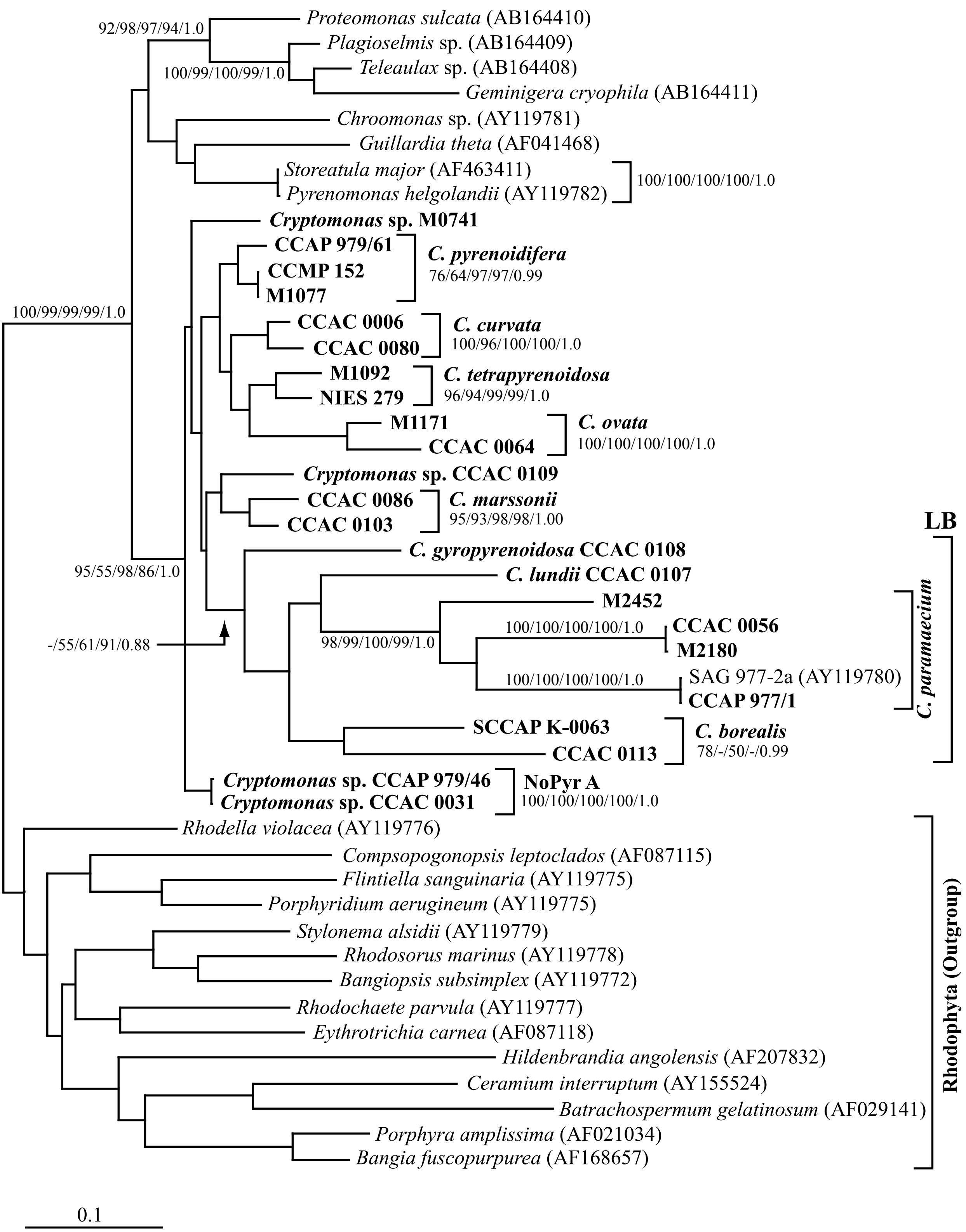

Additional file 3 - Rooted maximum likelihood tree of cryptophyte *rbc*L sequences including all codon positions (46 taxa; 990 nt)

As outgroup taxa, 14 rhodophyte *rbc*L sequences representing most bangiophyte clades and the Florideophyceae were chosen. In this tree, clade LB is present, but not always supported. Support values from left to right: maximum likelihood bootstrap/maximum parsimony bootstrap/distance bootstrap/logdet bootstrap/posterior probabilities (Bayesian analysis). Evolutionary model according to the Akaike Information Criterion: GTR+I+Γ. Bold face, new sequences; scale bar, substitutions per site. -*ln*L = 14480
