# Supplementary material for: Lineage-specific variations of congruent evolution among DNA sequences from three genomes, and relaxed selective constraints on rbcL in Cryptomonas (Cryptophyceae)
Source: BMC Evol Biol. 2005 Oct 18;5:56. doi: 10.1186/1471-2148-5-56 (PMC1285359; doi:10.1186/1471-2148-5-56)
Supplement: Additional File 4 — Rooted maximum likelihood tree of concatenated nuclear SSU rDNA, partial nuclear LSU rDNA, nucleomorph SSU rDNA and rbc L sequences [file 1471-2148-5-56-S4.pdf]

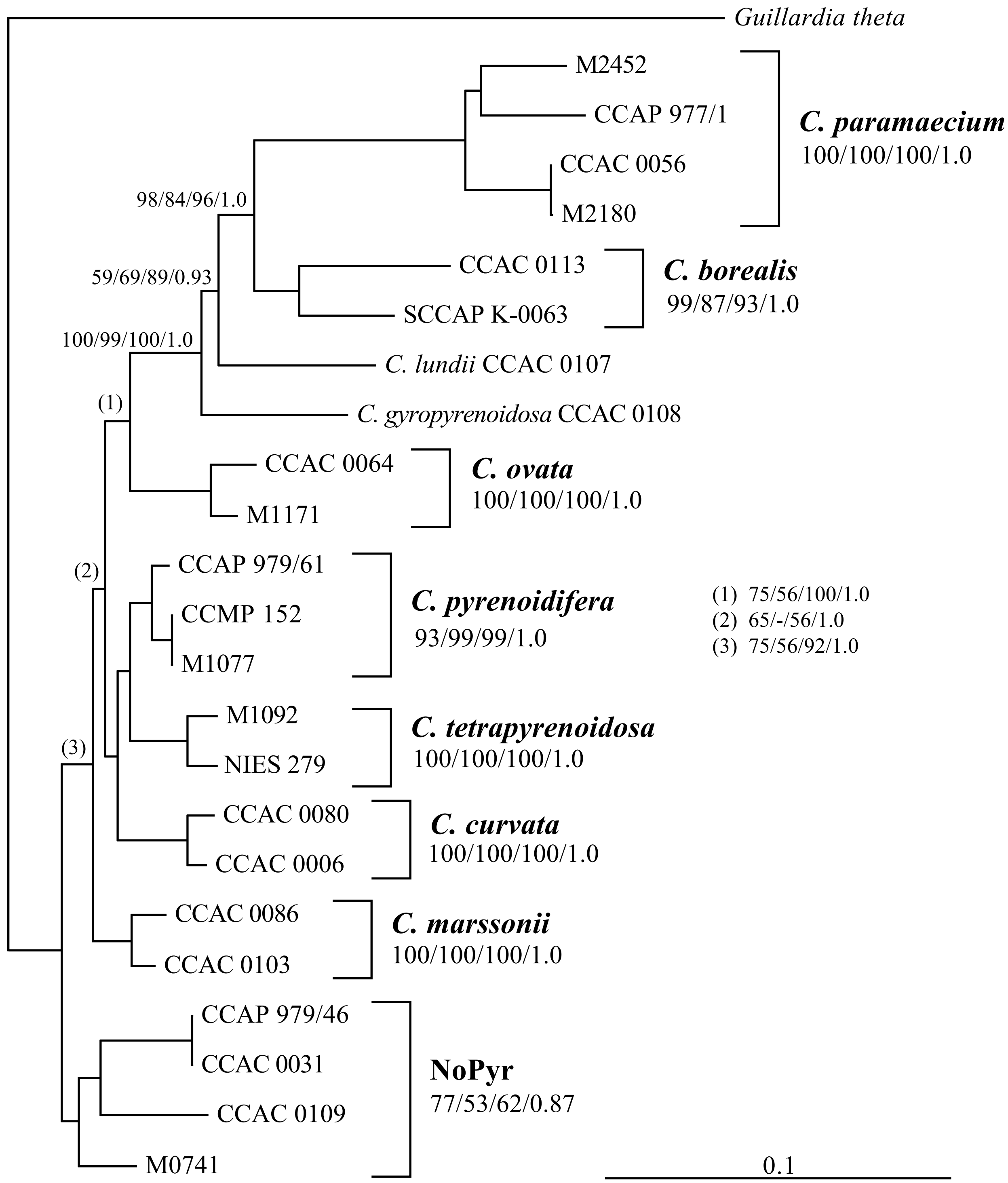

Additional file 4 - Rooted maximum likelihood tree of concatenated nuclear SSU rDNA, partial nuclear LSU rDNA, nucleomorph SSU rDNA and *rbc*L sequences

Twenty-four taxa; 5154 positions; for Bayesian analysis, the data set was subdivided into four partitions (nuclear LSU rDNA, 996 positions; nuclear SSU rDNA, 1536 positions; nucleomorph SSU rDNA, 1464 positions; *rbc*L, 1188 positions); likelihood settings were set to GTR+I+Γ+covarion with unlinked state frequencies, shape parameter, rate matrix and covarion switch rates. Support values from left to right, maximum likelihood bootstrap/ maximum parsimony bootstrap/ distance (neighbor-joining) bootstrap/ posterior probabilities (Bayesian analysis). Evolutionary model, GTR+I+Γ; scale bar, substitutions per site; -*ln*L = 21714
